# Does necroptosis have a crucial role in hepatic ischemia-reperfusion injury?

**DOI:** 10.1371/journal.pone.0184752

**Published:** 2017-09-28

**Authors:** Waqar K. Saeed, Dae Won Jun, Kiseok Jang, Yeon Ji Chae, Jai Sun Lee, Hyeon Tae Kang

**Affiliations:** 1 Department of Internal Medicine, Hanyang University School of Medicine, Seoul, South Korea; 2 Department of Translational Medicine, Hanyang University Graduate school of Biomedical Science and Engineering, Seoul, South Korea; 3 Department of Pathology, Hanyang University School of Medicine, Seoul, South Korea; Faculty of Medicine & Health Science, UNITED ARAB EMIRATES

## Abstract

**Background:**

Previous studies have demonstrated protective effects of anti-receptor interacting protein kinase 1 (RIP1), a key necroptosis molecule. However, it is uncertain whether necroptosis has a crucial role in hepatic IR injury. Therefore, we evaluated the role of necroptosis in hepatic IR injury.

**Method:**

The IR mice underwent 70% segmental IR injury induced by the clamping of the hepatic artery and portal vein for 1 hr followed by reperfusion for 4 hr. The key necroptosis molecules (RIP1, RIP3, and MLKL) and other key molecules of regulated necrosis (PGAM5 and caspase-1) were evaluated in the warm IR injury model. A RIP1 inhibitor (necrostain-1s) and/or an anti-mitochondrial permeability transition (MPT)-mediated necrosis mediator (cyclosporine A, CyA) were administered before clamping. Necrotic injury was quantified using Suzuki’s scoring system. qRT-PCR and western blot were performed to evaluate RIP1, RIP3, MLKL and PGAM5 expressions.

**Results:**

RIP1, RIP3, MLKL and PGAM5 expression did not change in the hepatic IR injury model. Moreover, Nec1s pretreatment did not improve histology or biochemical markers. The overall Suzuki score (cytoplasmic vacuolization, sinusoidal congestion and hepatocytes necrosis) was increased in the RIP3^(-/-)^ mice compared to the IR group (3.5 vs. 5, p = 0.026). CyA pretreatment and/or RIP3^(-/-)^ mice decreased Bax/Bcl2 expression; however, it did lead to an overall change in the levels of AST, ALT and LDH or necrotic injury. The Bax/Bcl2 ratio and the expression of caspase-1 and caspase-3 did not increase in our hepatic IR injury model.

**Conclusion:**

Key necroptosis molecules did not increase in the necrosis-dominant hepatic IR injury model. Anti-necroptosis and/or cyclosporine-A treatment did not have an overall protective effect on necrosis-dominant hepatic IR injury.

## Introduction

Hepatic ischemia-reperfusion (IR) injury is a well-recognized clinical concern during transplantation, septic shock and tumor resection [1]. Although both necrosis and apoptosis have a role in hepatic IR injury, necrosis seems to possess a dominant role in hepatic IR injury [2, 3].

Necroptosis is a newly defined cell death pathway mediated by specific molecular machinery requiring receptor-interacting protein kinase-1 (RIPK1), RIPK3, and mixed lineage kinase domain-like protein (MLKL) [4, 5]. Recently, several studies have demonstrated the protective effects of anti-necroptosis approach in a number of ischemia-reperfusion injury models, such as retinal IR [6], myocardial infarction [7], renal IR injury[8], and neonatal brain hypoxia-ischemia [9, 10]. Similarly, the protective role of anti-MPT-mediated necrosis by cyclophilin-D (Cyp-D) inhibition has also been suggested in the regulation of necrotic but not apoptotic cell death, as Cyp-D-deficient mice exhibited increased resistance to cardiac [11] and renal ischemia-reperfusion injuries [8]. Moreover, a number of studies have reported the protective effects of RIP1 and RIP3 inhibition in various liver disease models, such as non-alcoholic fatty liver disease, alcoholic liver disease, and toxic hepatitis model [12–14]. However, little is known about the role of regulated necrosis in hepatic IR.

Although necrostatin-1 (anti-RIP1 molecule) treatment has shown organ-protective effects in various IR injury models, except in the liver [6, 7, 10, 15], there are only two studies using a hepatic IR model [16, 17]. Interestingly, the results of the two previous studies were opposite. Necrostatin-1 did not attenuate IR injury induced leukocyte migration, perfusion failure, and hepatocellular injury in Rosentreter’s study [16]. However, liver histology was worsened and RIP1/RIP3 necrosome formation was attenuated by necrostain-1s in Hong’s study. Moreover, neither study evaluated the anti-RIP3 effects on the hepatic IR model. Until recently, RIP3 and MLKL were thought to represent key molecules of necroptosis-related cell death pathway. Initially, RIP1 was thought to be the key molecule of the necroptosis pathway; however, several alternative or RIP-1-independent pathways have been demonstrated.

Therefore, the role of regulated necrosis in hepatic IR is still unknown. We, therefore, investigated the role of regulated necrosis in hepatic IR injury. First, we administered Nec-1s, and used RIP3^(-/-)^ mice to evaluate the effects of the necroptosis pathway on hepatic IR injury. Later, we administered cyclosporine-A with Nec1s to wild type (WT) and RIP3^(-/-)^ animals to evaluate their combined effect on hepatic IR injury.

## Materials and methods

### Animal study design

Male C57BL/6 mice were obtained from the Orient Animal Laboratory (Seoul, South Korea), and RIP3^−^/^−^ mice were obtained from Genentech (San Francisco, CA, USA). The mice were maintained (4 per cage) in a temperature-controlled room (22°C) on a 12:12 h light-dark cycle. The mice were randomly divided into the following four groups (n = 8): sham, IR, IR+Nec1s (BioVision Milpitas, CA, USA) and IR injury in RIP3^−^/^−^ mice (IR+RIP3^−^/^−^). The genotype of the RIP3^−^/^−^ mice were confirmed using the primer sequences shown in S1 Fig. A non-lethal model of segmental (70%) hepatic warm ischemia was established as previously described [1], with minor modifications. Briefly, the animals were anesthetized with zoletil and rompun (1:1) i.p. A midline incision was made without damaging the adjacent structures. Ischemia was induced by clamping the hepatic artery and portal vein with an atraumatic clip for 60 min. The induction of ischemia was visually confirmed by change in the color of the liver from brownish red to pale yellow. The animals were kept warm by keeping them under a heat lamp, and the abdomen was wrapped with a plastic sheet to avoid excessive dehydration. After 60 min, blood flow was restored, and the abdomen was closed with double suturing. Nec1s (1.65 mg/kg body weight in 2% DMSO, i.p) was given 15 min before the clamping. Sham animals also underwent an identical surgical procedure without clamping. Sham and IR groups also received equal volumes of DMSO. Animals were euthanized by cutting the diaphragm under anesthesia 4 hr after reperfusion, and serum and liver samples were collected. All experiments were approved by the Hanyang Institutional Animal Care and Use Committee (HY-IACUC-15-0085, and HY-IACUC-13-0137).

In the second in vivo experiment, the mice were divided into the following five groups (n = 8): sham, IR, IR+CyA (BioVision Milpitas, CA, USA), IR+CyA+Nec1s and RIP3^−^/^−^+CyA to evaluate the role of cyclosporine administration in hepatic IR injury. Cyclosporine A (30 mg/kg in 150 μl of olive oil, i.p) was administered 24 hr before the IR injury. The sham and IR groups also received equal volumes of olive oil.

### Biochemical analysis

After 4 hr of reperfusion, the blood samples were withdrawn via cardiac puncture. Whole blood samples were collected in BD serum separation tubes, stored on ice and centrifuged at 3,000 rpm for 10 min. The serum samples were collected and stored at -70°C until analysis. Serum alanine aminotransferase (ALT), aspartate aminotransferase (AST) and lactate dehydrogenase (LDH) were measured with an automatic chemical analyzer (Hitachi-747; Hitachi, Tokyo, Japan).

### Histological analysis

Paraformaldehyde-fixed, paraffin-embedded liver tissue samples were sectioned (4 μm) and stained with hematoxylin and eosin for microscopic analysis. Images were obtained using a Leica DM4000B microscope. An experienced pathologist evaluated the sections, and the histological severity of the liver damage was graded using Suzuki’s score. It comprised of three components (sinusoidal congestion, hepatocyte necrosis, and ballooning degeneration), which were graded from 0 to 4.

### Western blot analysis

For western blot analysis, snap-frozen liver tissue samples were washed with normal saline. The tissues were homogenized using PRO-PREP Protein Extraction Solution (iNtRON Biotechnology, FL, USA). The protein extracts were quantified, and 25 μg of protein was transferred to sample buffer, separated by 10% sodium dodecyl sulfate polyacrylamide gel electrophoresis and transferred to PVDF membranes (Immobilon-P; Millipore, Billerica, MA, USA). After blocking with 5% BSA solution for 1 hr, the membranes were incubated with primary antibodies against RIP1, RIP3, MLKL, and PGAM5 (Abcam, Cambridge, MA, USA) as well as β-actin (Santa Cruz Biotechnology, Santa Cruz, California) followed by incubation with secondary antibodies. The bands were visualized with West-Q Chemiluminescent Substrate Kit Plus (GenDEPOT, TX, USA). The results were obtained with an image analyzer (Image lab 3.0, Bio-Rad, Hercules, CA, USA) and were quantified using ImageJ (http://imagej.nih.gov/ij).

### Quantitative real-time polymerase chain reaction (qRT-PCR)

Total RNA was extracted from liver tissue using TRIzol reagent (Invitrogen, Carlsbad, USA) according to the manufacturer’s instructions. RNA concentrations were measured using a Nanodrop ND-2000 spectrophotometer (Thermo Fisher Scientific Inc., DE, USA), and the purity was determined by measuring the A260/A280 ratio. First-strand cDNA was synthesized using the PrimeScript 1st Strand cDNA Synthesis Kit (Takara Bio, Japan). Quantitative real-time polymerase chain reaction (qRT-PCR) was performed using the LightCycler 480 SYBR Green I Master Mix (Roche Diagnostics, IN, USA) and analyzed on a LightCycler 480 II System (Roche Diagnostics, Indianapolis, IN, USA). qPCR amplification was performed with incubation for 5 min at 95°C followed by 45 cycles of 10 s at 95°C, 10 s at 60°C, and 10 s at 72°C and a final dissociation step at 65°C for 15 s. The crossing point of each sample was automatically determined by the LightCycler, and the relative change ratio was determined using the ratio of mRNA for the selected gene and that for glyceraldehyde-3-phosphate dehydrogenase (GAPDH). PCR transcript levels were normalized against those of GAPDH. The PCR primer sets used were as follows: mouse GAPDH forward, 5/- AGAAACCTGCCAAGTATGATG-3/ and reverse, 5/-GGAGTTGCTGTTGAAGTCG-3/; mouse RIP1 forward, 5/- GAAGGCATGTGCTACTTACATGACA-3/ and reverse, 5/- TAATGTGAAAGTCACGATCAACGAG-3/; mouse RIP3 forward, 5/- AGAACTGAAGAAGCTGGAGTTTGTG-3/ and reverse, 5/- ATCTTGACTGCTACATCATGGTTCC-3/; mouse MLKL forward, 5/- TATGTCTCCCCTGAGAGACTGAAAA-3/ and reverse, 5/- TTCCCAGAGTACAATTCCAAAGCTA-3/; PGAM5 forward, 5/- CTTGCAAGCCTGGGATTAAAGTT -3/ and reverse, 5/- TGCTGATGATGTCTGTGGTCTCTAC-3/; Bax forward, 5/-AGATGAACTGGACAGCAATATGGAG-3/ and reverse, 5/- ACCCGGAAGAAGACCTCTCG-3/; and Bcl2 forward, 5/- TGTGGATGACTGAGTACCTGAACC-3/ and reverse, 5/- TCATTCAACCAGACATGCACCTAC-3.

### Statistical analysis

The values were expressed as the mean ± SD and were obtained from three independent replicates. Statistical analyses were performed using SPSS version 18.0 for Windows (SPSS Inc., Chicago, IL, USA). One-way analysis of variance and independent t-tests were performed to compare the means of different values. A *p-*value of less than 0.05 was considered statistically significant.

## Results

### Receptor-interacting protein kinase-1 in the hepatic ischemia-reperfusion injury model

**Fig 1:**
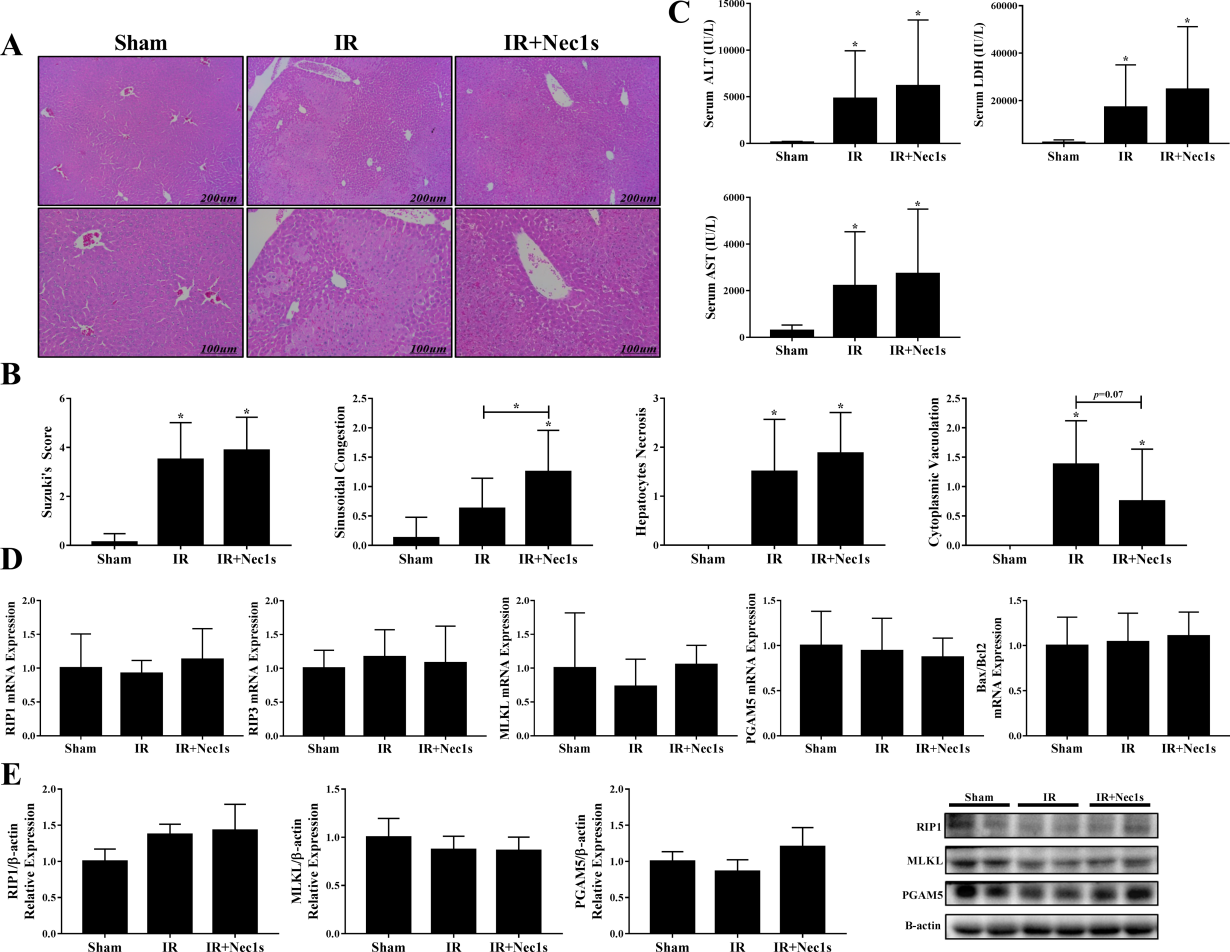
Effects of anti-receptor interacting protein 1 (RIP1) treatment on hepatic ischemic-reperfusion injury model. (A&B) H&E staining and Suzuki score of the sham, IR and Nec1s groups. Following IR injury, the Suzuki score increased; however, the Suzuki score between the IR and Nec1s groups was not different. (C) Changes in serum AST, ALT and LDH levels following IR injury. (D) RIP1, RIP3, MLKL and PGAM5 expression among the groups. We did not found a significant difference in RIP1, RIP3, MLKL and PGAM5 expression among the groups. Bax/Bcl2 expression among the groups. (E) Western blot analysis of RIP1, MLKL and PGAM5 protein expression among the groups.

Hematoxylin and eosin staining showed significant necrotic injury in the IR group compared to the sham group (Fig 1A); however, a significant decrease in necrotic injury was not observed in the Nec1s pretreatment group compared to the IR group. The extent of hepatic damage was quantified using Suzuki’s score, which evaluates sinusoidal congestion, vacuolization and necrosis on a four-point scale for a total score of 0–12 (Fig 1B). Serum AST (2,203 vs. 2,722 IU/L, *p* = 0.691), ALT (4,803 vs. 6,165 IU/L, *p* = 0.680), and LDH (17,221 vs. 24,757 IU/L, *p* = 0.515) did not change in the IR+Nec1s group compared to the IR group (Fig 1C). RIP1, RIP3, MLKL and PGAM5 expressions were evaluated to assess the extent of the changes in key necroptosis signaling molecules following IR injury. However, we did not find any statistically significant change in RIP1, RIP3, MLKL and PGAM5 expression between the sham, IR and Nec1s pretreatment groups (Fig 1D and 1E).

### Receptor-interacting protein kinase- 3 in the hepatic ischemia-reperfusion injury model

**Fig 2:**
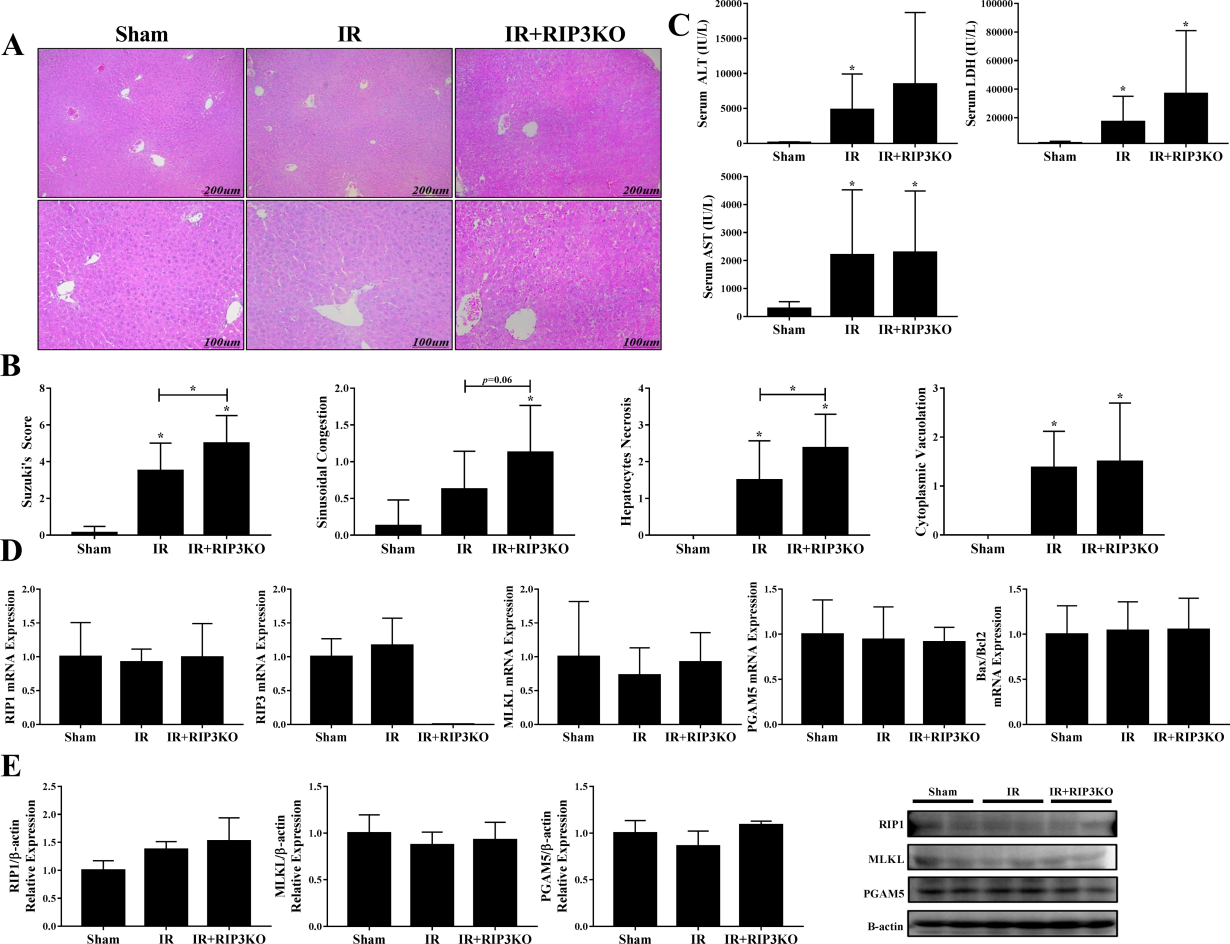
Role of receptor interacting protein 3 (RIP3) in hepatic ischemic-reperfusion injury model. (2A&B) H&E staining and Suzuki score of the sham, IR and RIP3-/- groups. Following IR injury, the Suzuki score increased; however, interestingly, the RIP3-/- group exhibited an increased Suzuki score compared to the IR group. (C) Changes in serum AST, ALT and LDH levels among the sham, IR and RIP3-/- groups. (D) RIP1, RIP3, MLKL and PGAM5 expressions among the sham, IR and RIP3-/- groups. We found no significant difference in RIP1, MLKL and PGAM5 expression among the groups. Bax/Bcl2 expression among the sham, IR and RIP3-/- groups. (E) Western blot analysis of RIP1, MLKL and PGAM5 protein expression among the sham, IR and RIP3-/- groups.

Next, we used RIP3^(-/-)^ mice to evaluate whether RIP3 is involved in hepatic IR injury. Hematoxylin and eosin staining showed increased hepatic injury in RIP3^(-/-)^ mice compared to the IR group (Fig 2A). The overall Suzuki score was increased in RIP3^(-/-)^ mice compared to the IR group (3.5 vs. 5, *p* = 0.026). Cytoplasmic vacuolization (1.37 vs. 1.5, p = 0.761), sinusoidal congestion (0.62 vs. 1.12, *p* = 0.06) and hepatocytes necrosis (1.5 vs. 2.3, *p* = 0.043) were all increased in RIP3^(-/-)^ mice compared to the IR group (Fig 2B). However, no significant difference in serum AST (2,203 vs. 2,292 IU/L, *p* = 0.941), ALT (4,803 vs. 8,457 IU/L, *p* = 0.628) and LDH (17,221 vs. 36,738 IU/L, *p* = 0.276) levels between the IR and RIP3^(-/-)^ groups was observed (Fig 2C). Moreover, similar to the results of Nec1s administration, there was no statistically significant difference in RIP1, MLKL and PGAM5 expressions among the groups (Fig 2D and 2E).

### Combined cyclosporine-A administration and necroptosis inhibition

**Fig 3:**
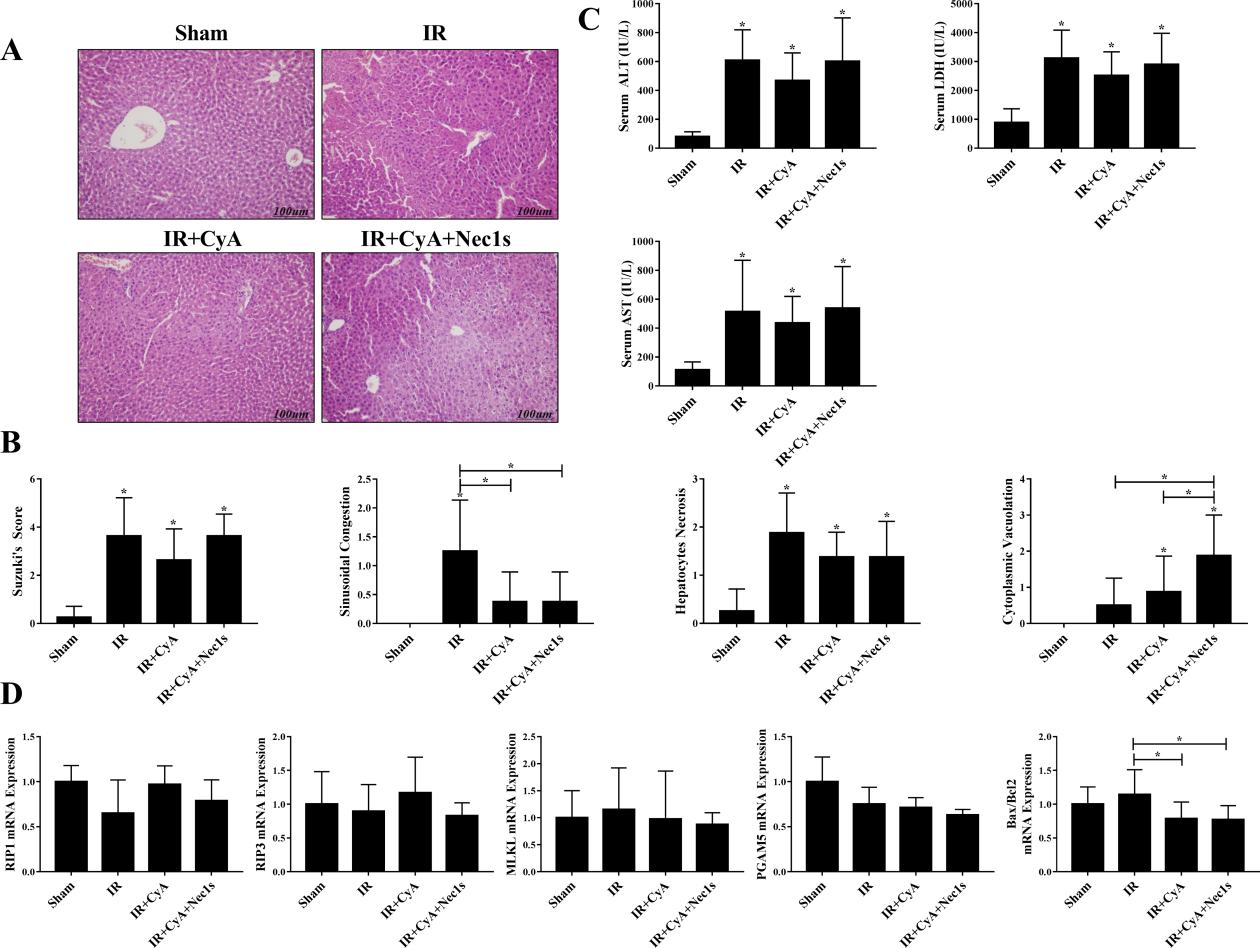
Effects of co-treatment with anti-RIP1 and cyclosporine on hepatic ischemic-reperfusion injury. (A&B) H&E staining and Suzuki score among the sham, IR, IR+CyA and IR+CyA+Nec1s groups. Although, the overall Suzuki score increased following IR injury, there was no significant difference in Suzuki score among the treatment groups. (C) Changes in serum AST, ALT and LDH levels among the sham, IR, IR+CyA and IR+CyA+Nec1s groups. (D) RIP1, RIP3, MLKL and PGAM5 expression among the groups. Although, the Bax/Bcl2 ratio decreased following CyA administration, no significant difference in RIP1, RIP3, MLKL and PGAM5 expressions was observed.

**Fig 4:**
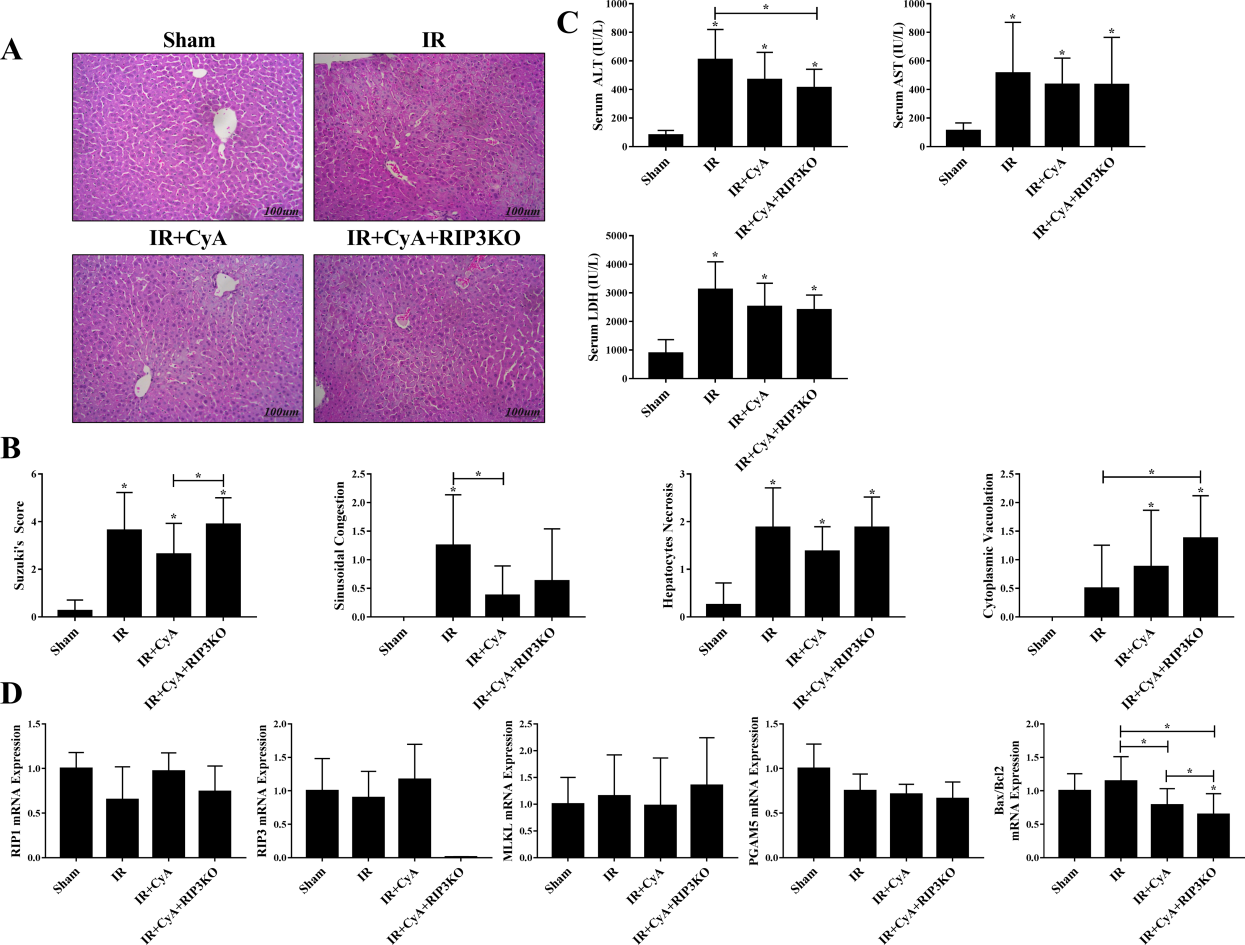
Effects of cyclosporine in RIP3-/- mice in hepatic ischemic-reperfusion injury model. (A&B) H&E staining and Suzuki score among the sham, IR, IR+CyA and IR+CyA+RIP3-/- groups. Although, the overall Suzuki score increased following IR injury, interestingly, the IR+CyA+RIP3-/- group had the highest score among the groups. (C) Changes in serum AST, ALT and LDH levels among sham, IR, IR+CyA and IR+CyA+RIP3-/- groups. (D) RIP1, RIP3, MLKL, PGAM5, and Bax/Bcl2 expressions among the groups.

We evaluated the effects of anti-mitochondrial permeability transition (MPT)-mediated necrosis mediator (cyclosporine A, CyA) mono-treatment and combined administration with anti-necroptotic treatment (CyA combined with Nec1s or RIP3^(-/-)^) in hepatic IR injury. Although sinusoidal congestion was decreased in the IR+CyA, IR+CyA+Nec1s, IR+CyA+RIP3^(-/-)^ groups compared to the IR group; the overall extent of hepatic injury as determined by the Suzuki’s scoring system did not improve in either the IR+CyA+Nec1s (Fig 3A and 3B) or IR+CyA+RIP3^(-/-)^ groups (Fig 4A and 4B). CyA administration decreased Bax/Bcl2 expression following IR injury; however, it did not significantly improve the overall histology or hepatic injury serum marker expression (Figs 3D and 4D). Interestingly, the CyA+RIP3^(-/-)^ group had an increased overall Suzuki’s score compared to the IR+CyA group (2.6 vs. 3.8, *p* = 0.046) (Fig 4B).

## Discussion

Our data showed that the regulated necrosis machinery was not activated in hepatic IR injury. Necroptosis (the RIP1 and 3 pathways) and the MPT-mediated necrosis pathway did not have a crucial role in hepatic IR injury. This is the first study to extensively investigate the evidence and the role of programed necrosis in hepatic IR.

Although necrostatin-1 treatment showed organ-protective effects in various IR injury models except the liver [6, 7, 10, 15], our data demonstrated that nec-1 (data not shown) and nec-1s (stable long acting nec-1) did not exhibit protective effects on the hepatic IR injury model. This finding was somewhat different from Hong’s data [17] but was similar to another study [16]. However, none of the previous IR injury studies extensively evaluated changes in the key regulated necrosis machinery (RIP1, RIP3, MLKL, and PGAM5) or the effect of RIP3 inhibition in their IR model. This is the first study to evaluate the possible role of necroptosis in hepatic IR injury. Initially, RIP1 was thought to be the key molecule of the necroptosis pathway; however, until recently, RIP3 and MLKL were thought to represent key molecules of the necroptosis-related cell death pathway.

**Fig 5:**
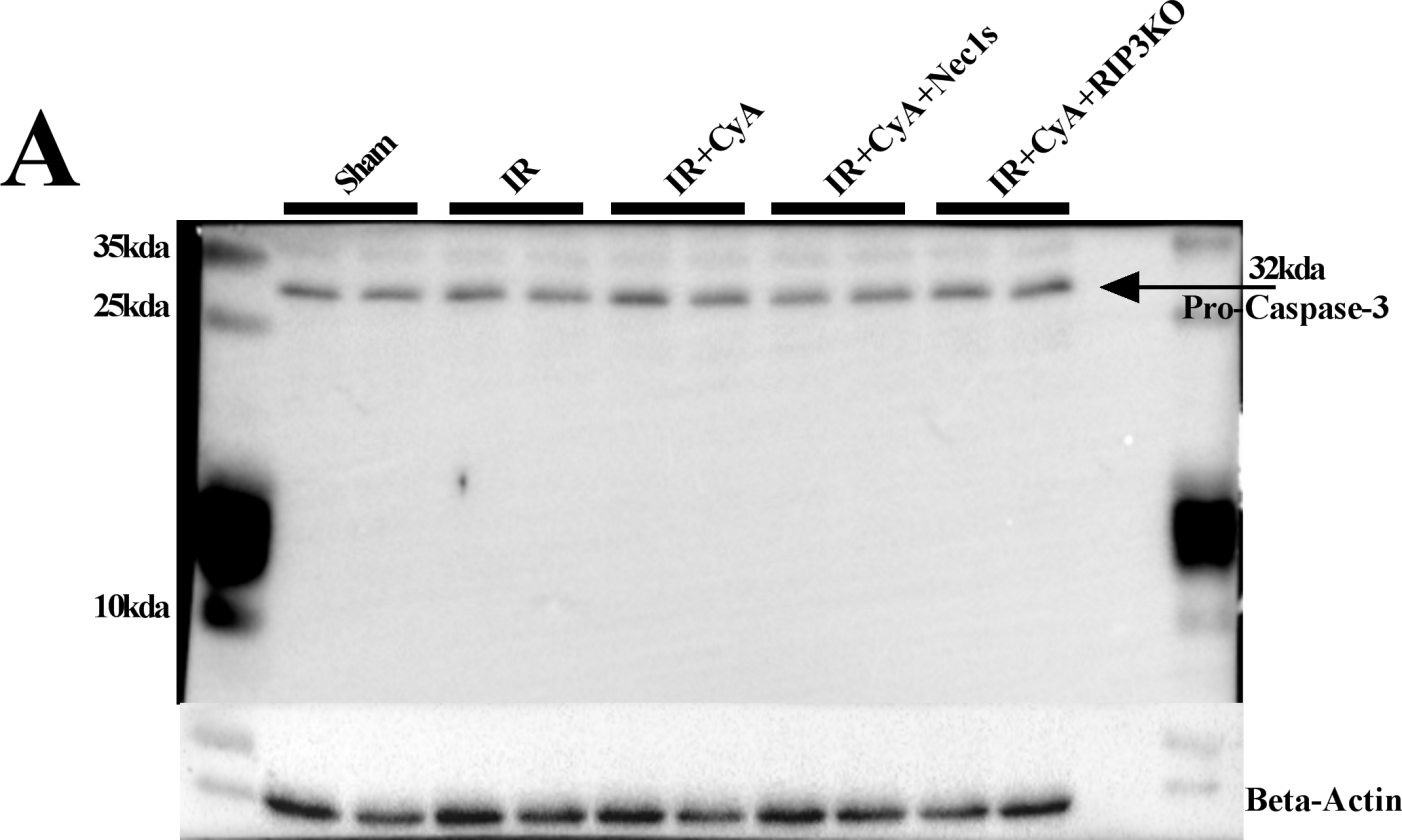
Caspase-3 expression among the groups. Caspase-3 expression did not increased following IR injury. Our results showed that RIP1/RIP3 inhibition and the anti-mitochondrial permeability transition-mediated necrosis mediator (cyclosporine A, CyA) did not attenuate hepatic IR injury. It is quite natural, because expression of the key regulated necrosis machinery did not change in our necrosis-dominant IR model.

Our data regarding the effects of CyA administration on hepatic IR injury were somewhat different compared to that of previous studies. It seemed to be affected by the type of hepatic IR model. The effects of CyA depend on the type of IR injury (apoptosis dominant vs necrosis dominant). There are two types of warm hepatic IR injury models: the so-called apoptosis dominant and necrosis dominant models. Previously, two studies have evaluated the protective effects of cyclosporine-A administration on hepatic ischemia-reperfusion injury. Focusing on apoptosis in a rat model of warm hepatic IR injury, cyclosporine significantly reduced apoptosis and neutrophil infiltration [18]. However, another hepatic IR injury study using a necrosis-dominant IR injury model did not show an overall protective effect of Cyp-A on hepatic IR injury [19]. The characteristics and pathophysiology of hepatic IR injury are very different depending on the ischemic duration and degree. We adopted the necrosis-dominant hepatic IR injury model for the present study. The Bax/Bcl2 ratio and the expression of caspase-1 and caspase-3 expression did not significantly increase in our hepatic IR model (Figs 3D and 4D, Fig 5, S2 Fig).

Recently, evidences suggest necrosis dispensable role of PGAM5 and mitochondria [20]. Moreover, within the spectrum of regulated necrosis, several other pathways of regulated necrosis, such as the MPT-meditated regulated necrosis, pyroptosis, ferroptosis, and parthanatos. have also been suggested. Pyroptosis is a caspase-1-mediated cell death that exhibits necrotic morphology [4, 8]. Caspase-1 is a key molecule in pyroptosis, but caspase-1 expression did not increase in the hepatic IR model (S2 Fig). The involvement of mitochondrial permeability transition pore (MPT) via cyclophilin-D has been suggested for IR injury in other organs. Although combined CyA administration with an anti-RIP-1 or RIP-3 approach decreased Bax/Bcl2 expression, it did not have a significant overall protective effect in hepatic IR injury. The effects of anti-Cyp-D and RIP-3 treatment showed organ differences in IR injury.

There is an issue on non-specificity of commercially available RIP3 antisera. RIP3 was expressed after the ischemia-reperfusion injury even in the RIP3 KO mice (S1B Fig). Previous study also pointed out the same issue [21]. They used nine different commercially available RIP3 antisera. Most importantly all nine antibodies reacted with RIPK3 ^(-/-)^ liver indicating the non-specificity of the 55KD band seen with these antisera. In contrast, only monoclonal ‘Genentech antibody’ was highly sensitive and specific. We also tried several commercially available RIP3 antisera, the commercial available RIP3 antibody was detecting RIP3 band even in the RIP3 ^(-/-)^ mice.

Our study has the following limitations. First, our data cannot generalize all hepatic IR models because there are many hepatic IR models for instance, warm and cold IR injury models. Second, we did not use the MLKL^(-/-)^ mice. Recently, RIP3-independent and RIP1/MLKL-dependent necrosis have also been documented in human autoimmune and murine inflammation models [22]. Therefore, additional studies using MLKL^(-/-)^ mice would be needed. Third, we evaluated the expression of RIP1, and RIP3 using qRT-PCR and western blot. Immunoprecipitation for necrosome (RIP1-RIP3 complex), and using RIP1 kinase knockout mice will show more concrete evidence for role of RIP1 in hepatic IR injury. However, RIP1 knockout mice die at 1–3 days after birth and display extensive apoptosis in their lymphoid. And we did not try immunoprecipitation for necrosome because of non-specificity issues of commercial RIP3 antibody. ‘Genentech’ monoclonal antibody is not commercially available. Fourthly, for Nec1s, different time courses, dosage and administration routes should also be considered. There are some reports that Nec-1 or Nec-1s are effective just before the injuries or stimulations. In contrast, there are also some reports that these drugs need to be administered more than an hour before the events to show the protective effects. We administered Nec1s 15 min before clamping because of two reasons. First, earlier studies also administered Nec1 15 mins before clamping in animal experiments [8, 14, 23]. Second, in earlier studies, Nec1 was said to have a very short half-life [24]. Therefore, for further clarification and validation, a detailed and focused approach combining the use of several methods would be essential [25]; however, we only included one or two methods to draw the present conclusions. Therefore, specific genetic and pharmacological inhibition experiments are needed for further confirmation and re-evaluation.

In conclusion, the expression of key molecules of necroptosis did not increase in the necrosis-dominant hepatic IR model. Anti-necroptosis and/or cyclosporine-A treatment did not have an overall protective effect on necrosis-dominant hepatic IR. There is a need for further studies on necrosis component of hepatic ischemic reperfusion injury in human settings, so that the effective necrosis inhibition and thus organ survival could be achieved.

## Supporting information

S1 Fig Genotyping of RIP3-/- mice was performed by PCR using tail snips. RIP3 antibody reacting with RIP3-/- liver indicating non-specificity of the antibody.
(TIF)

S2 Fig Caspase-1 protein expression among the sham and IR animals. (TIF)

S1 File ARRIVE checklist. (DOCX)

S2 File Data.zip. (XLSX)

## Abbreviations

IR: Ischemic reperfusion
RIP1: Receptor interacting protein kinase-1
RIP1: Receptor interacting protein kinase-3
MLKL: Mixed lineage kinase domain-like protein
PGAM5: Phosphoglycerate mutase 5
CyA: Cyclosporine-A
ALT: Alanine aminotransferase
AST: Aspartate aminotransferase
LDH: Lactate dehydrogenase
