# Supplementary figures and images for: Does necroptosis have a crucial role in hepatic ischemia-reperfusion injury?
Source: PLoS One. 2017 Sep 28;12(9):e0184752. doi: 10.1371/journal.pone.0184752 (PMC5619711; doi:10.1371/journal.pone.0184752)

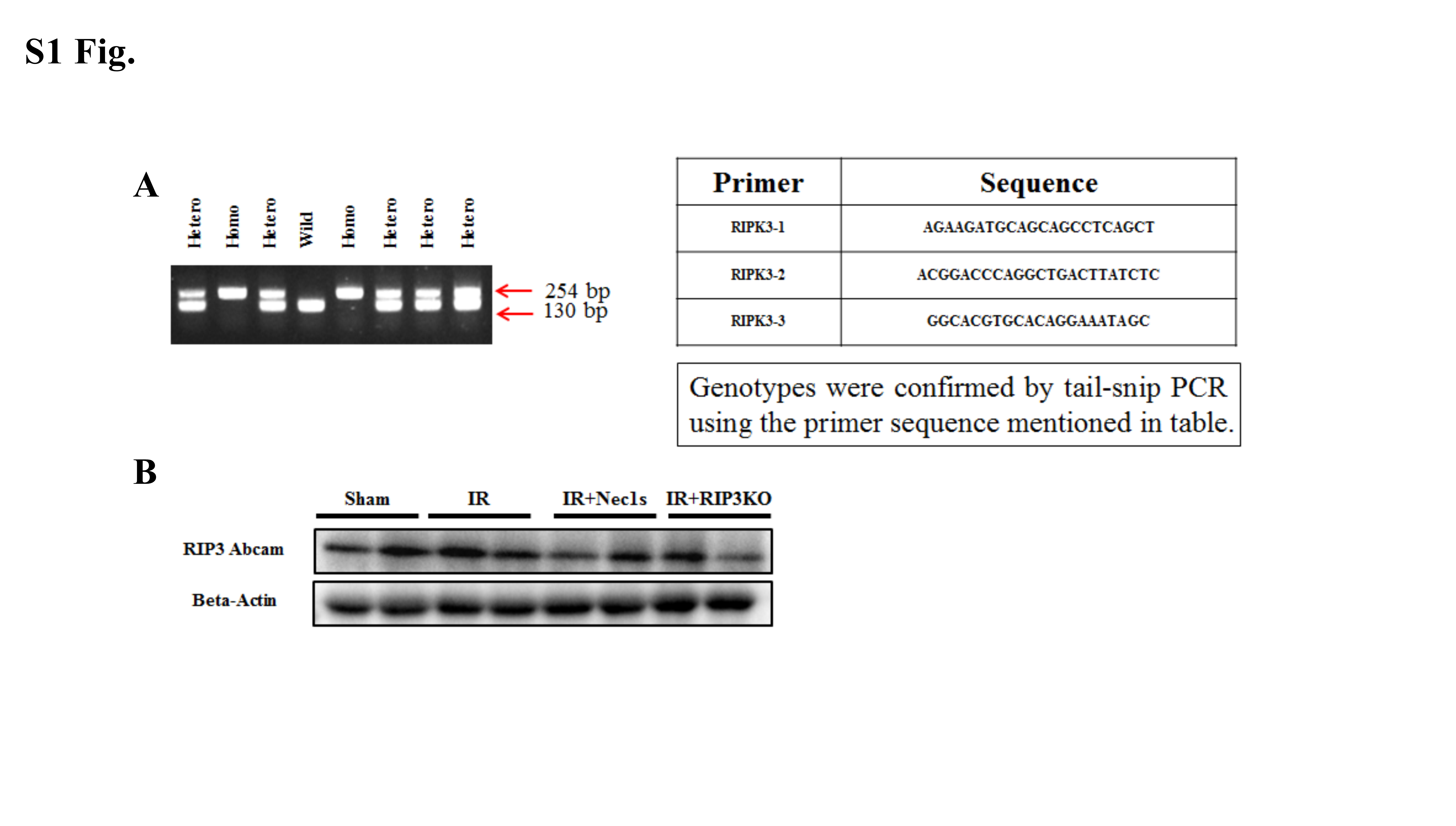

Supplement: S1 Fig — RIP3 antibody reacting with RIP3-/- liver indicating non-specificity of the antibody. (TIF) [file pone.0184752.s001.tif]

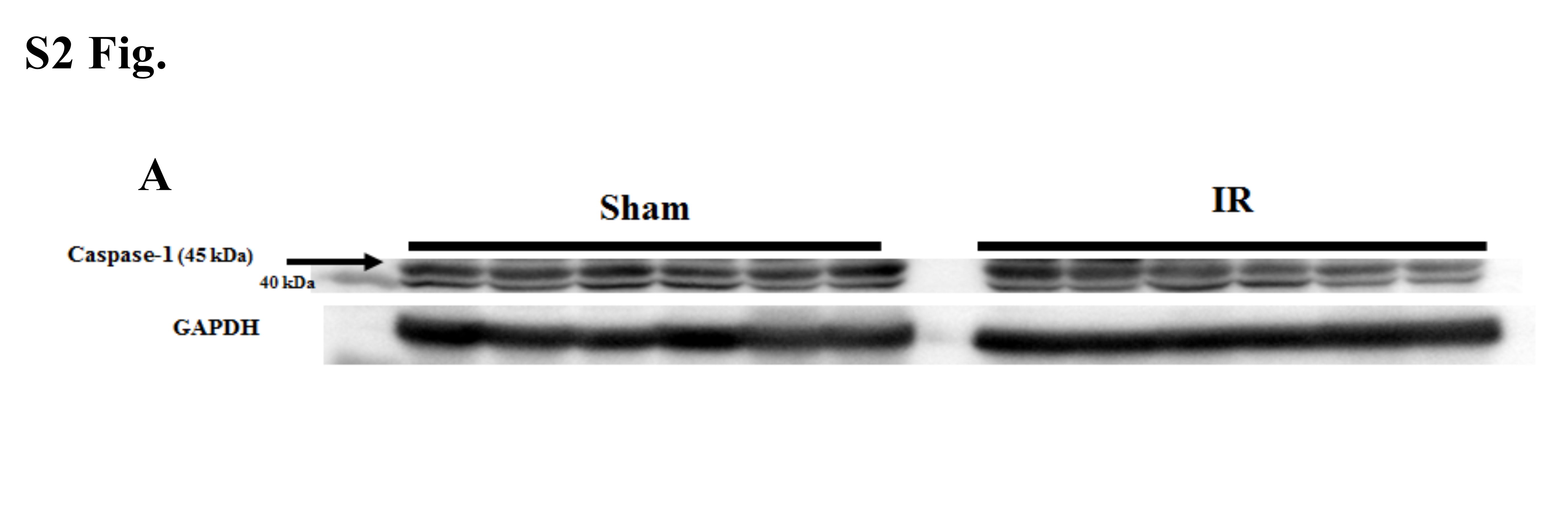

Supplement: S2 Fig — (TIF) [file pone.0184752.s002.tif]
