# Supplementary material for: Does necroptosis have a crucial role in hepatic ischemia-reperfusion injury?
Source: PLoS One. 2017 Sep 28;12(9):e0184752. doi: 10.1371/journal.pone.0184752 (PMC5619711; doi:10.1371/journal.pone.0184752)

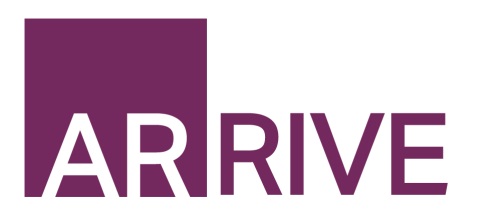


The ARRIVE Guidelines Checklist

Animal Research: Reporting In Vivo Experiments

Carol Kilkenny^1^, William J Browne^2^, Innes C Cuthill^3^, Michael Emerson^4^ and Douglas G Altman^5^

*^1^The National Centre for the Replacement, Refinement and Reduction of Animals in Research, London, UK, ^2^School of Veterinary Science, University of Bristol, Bristol, UK, ^3^School of Biological Sciences, University of Bristol, Bristol, UK, ^4^National Heart and Lung Institute, Imperial College London, UK, ^5^Centre for Statistics in Medicine, University of Oxford, Oxford, UK.*

|  | | ITEM | RECOMMENDATION | Section/ Paragraph |
| --- | --- | --- | --- | --- |
| 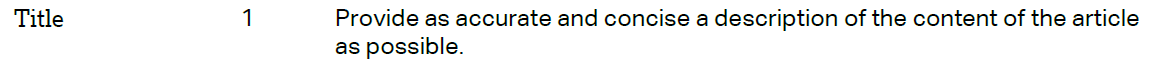 | | | Title |  |
| 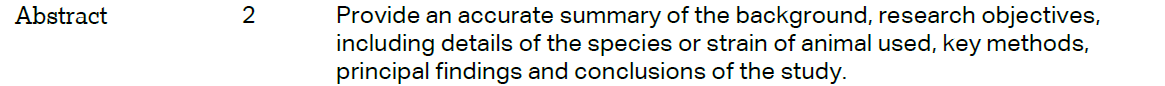 | | | Abstract |  |
| INTRODUCTION | | |  |  |
| 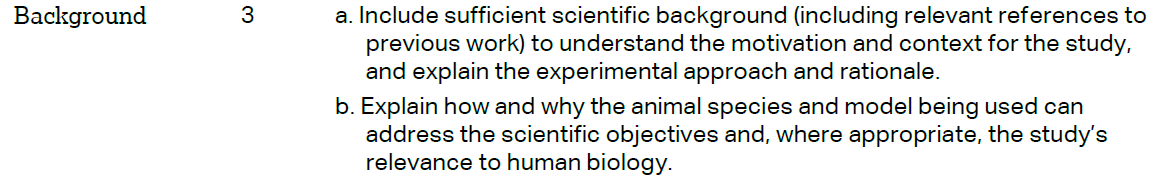 | | | Paragraph 1-4 |  |
| 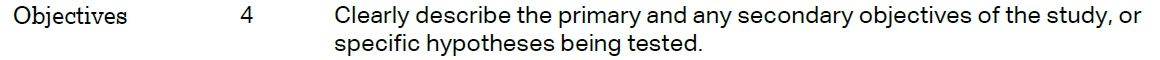 | | | Paragraph 4 |  |
| METHODS | | |  |  |
| 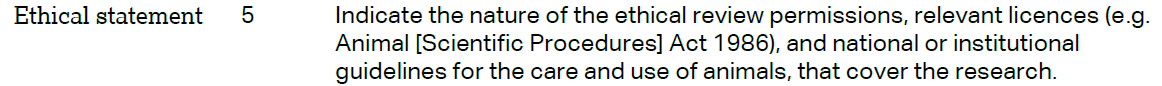 | | | Animal study design /Paragraph 1 |  |
| 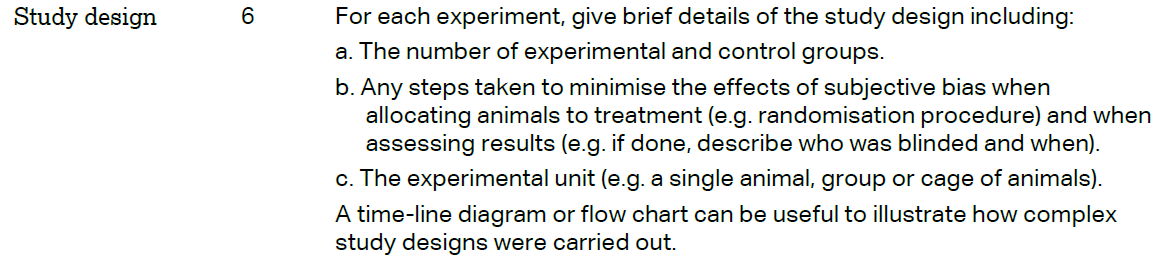 | | | Animal study design /Paragraph 1-2 |  |
| 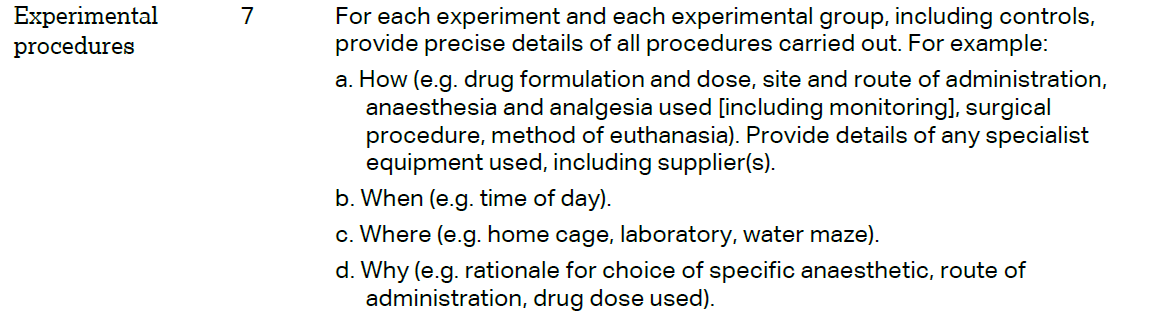 | | | Animal study design /Paragraph 1-2 |  |
| 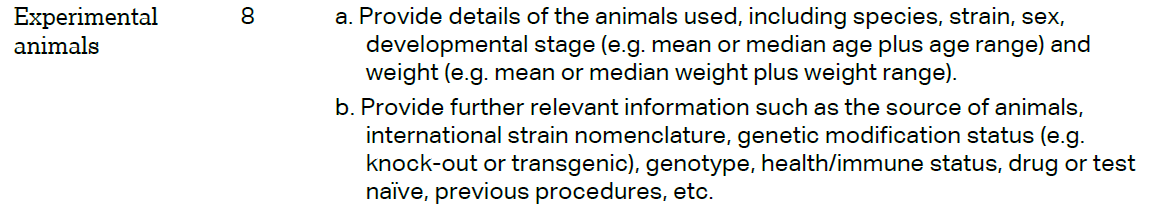 | | | Animal study design /Paragraph 1 |  |

The ARRIVE guidelines. Originally published in *PLoS Biology*, June 2010^1^

| 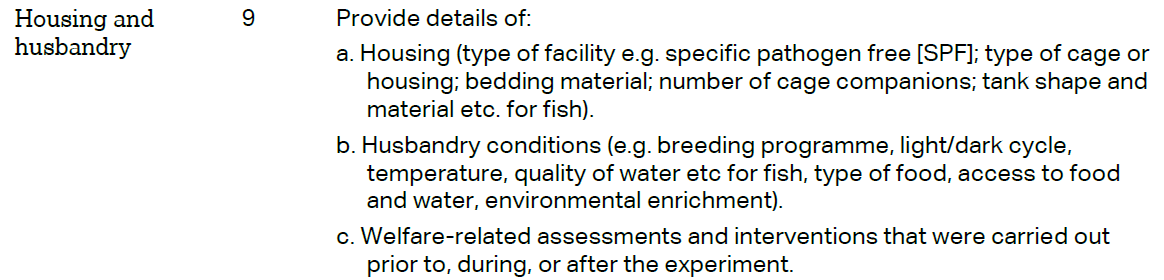 | Animal study design /Paragraph 1 | |
| --- | --- | --- |
| 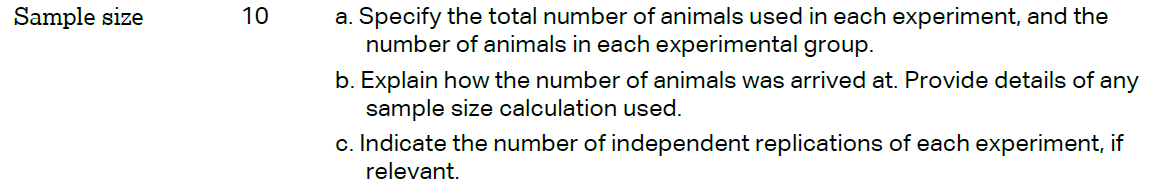 | Animal study design /Paragraph 1-2 | |
| 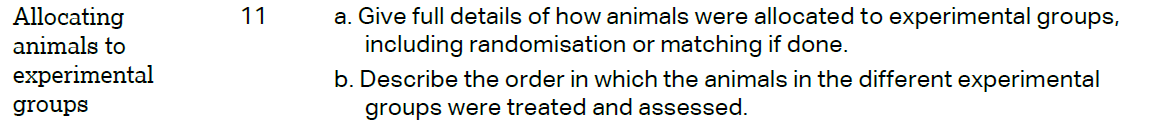 | Animal study design /Paragraph 1-2 | |
| 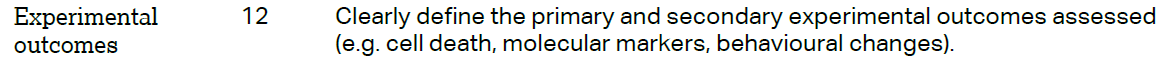 | Results/Subheading1-3/ Paragraph 1 | |
| 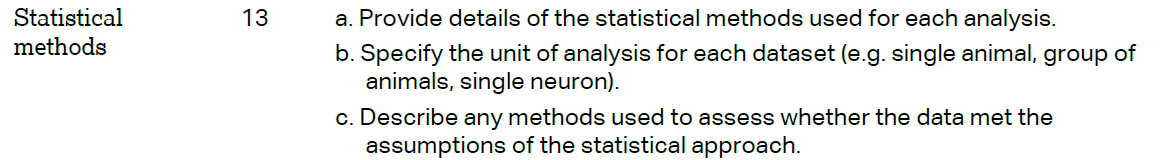 | Statistical Analysis/ Paragraph 1 | |
| RESULTS |  | |
| 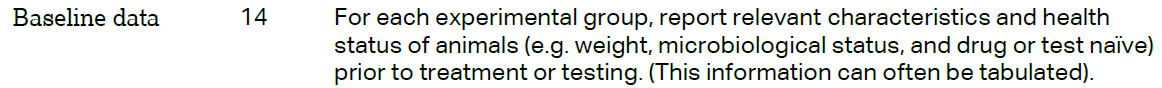 | Results/Subheading1-3/ Paragraph 1 | |
| 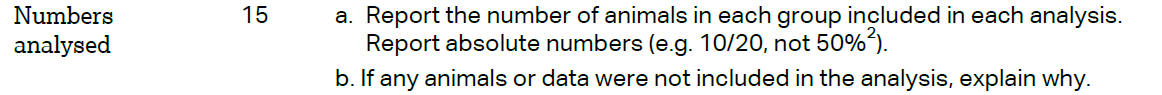 | Methods /Animal study design/ Paragraph 1 | |
| 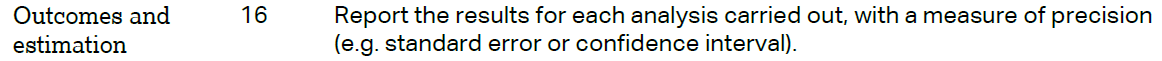 | Results/Subheading1-3/ Paragraph 1 | |
| 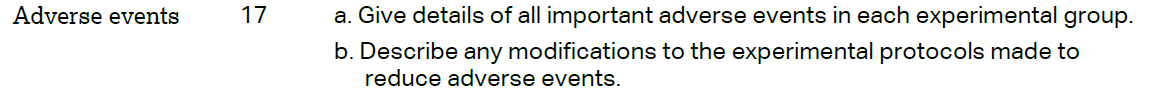 | Methods /Animal study design/ Paragraph 1 The animals were kept warm by keeping them under a heat lamp, and the abdomen was wrapped with a plastic sheet to avoid excessive dehydration. | |
| DISCUSSION |  | |
| 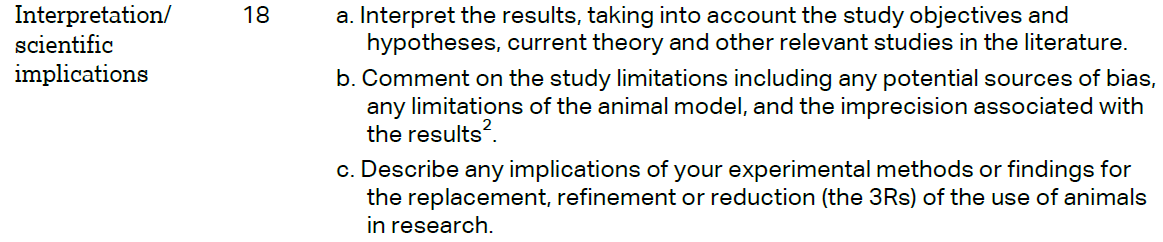 | Paragraph 1-8 | |
| 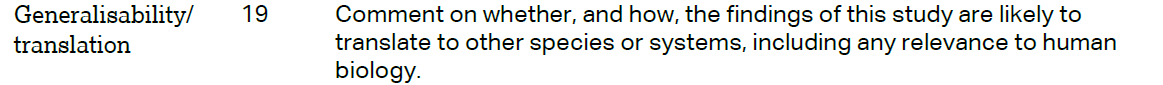 | Paragraph 6-8 | |
| 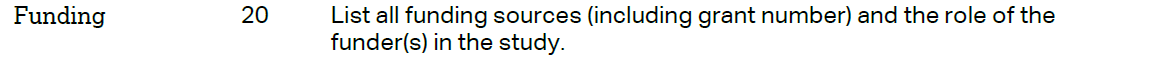 | | Page 2/Funding Information |


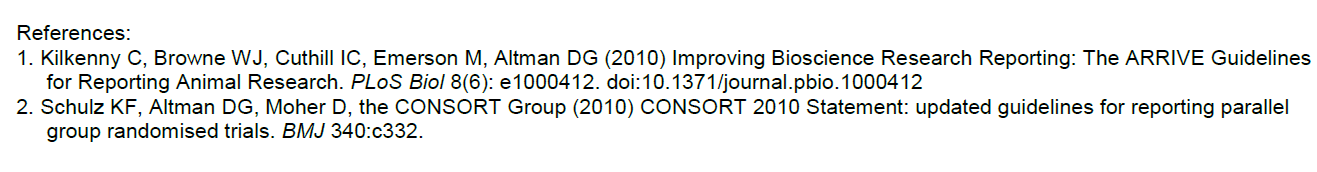

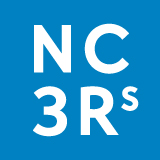

Supplement: S1 File — (DOCX) [file pone.0184752.s003.docx]
